# The PRISMA 2020 statement: An updated guideline for reporting systematic reviews

**DOI:** 10.1371/journal.pmed.1003583

**Published:** 2021-03-29

**Authors:** Matthew J. Page, Joanne E. McKenzie, Patrick M. Bossuyt, Isabelle Boutron, Tammy C. Hoffmann, Cynthia D. Mulrow, Larissa Shamseer, Jennifer M. Tetzlaff, Elie A. Akl, Sue E. Brennan, Roger Chou, Julie Glanville, Jeremy M. Grimshaw, Asbjørn Hróbjartsson, Manoj M. Lalu, Tianjing Li, Elizabeth W. Loder, Evan Mayo-Wilson, Steve McDonald, Luke A. McGuinness, Lesley A. Stewart, James Thomas, Andrea C. Tricco, Vivian A. Welch, Penny Whiting, David Moher

**Affiliations:** 1 School of Public Health and Preventive Medicine, Monash University, Melbourne, Australia; 2 Department of Clinical Epidemiology, Biostatistics and Bioinformatics, Amsterdam University Medical Centres, University of Amsterdam, Amsterdam, Netherlands; 3 Université de Paris, Centre of Epidemiology and Statistics (CRESS), Inserm, Paris, France; 4 Institute for Evidence-Based Healthcare, Faculty of Health Sciences and Medicine, Bond University, Gold Coast, Australia; 5 University of Texas Health Science Center at San Antonio, San Antonio, Texas, United States of America; *Annals of Internal Medicine*; 6 Knowledge Translation Program, Li Ka Shing Knowledge Institute, Toronto, Canada; School of Epidemiology and Public Health, Faculty of Medicine, University of Ottawa, Ottawa, Canada; 7 Evidence Partners, Ottawa, Canada; 8 Clinical Research Institute, American University of Beirut, Beirut, Lebanon; Department of Health Research Methods, Evidence, and Impact, McMaster University, Hamilton, Ontario, Canada; 9 Department of Medical Informatics and Clinical Epidemiology, Oregon Health & Science University, Portland, Oregon, United States of America; 10 York Health Economics Consortium (YHEC Ltd), University of York, York, United Kingdom; 11 Clinical Epidemiology Program, Ottawa Hospital Research Institute, Ottawa, Canada; School of Epidemiology and Public Health, University of Ottawa, Ottawa, Canada; Department of Medicine, University of Ottawa, Ottawa, Canada; 12 Centre for Evidence-Based Medicine Odense (CEBMO) and Cochrane Denmark, Department of Clinical Research, University of Southern Denmark, Odense, Denmark; Open Patient data Exploratory Network (OPEN), Odense University Hospital, Odense, Denmark; 13 Department of Anesthesiology and Pain Medicine, The Ottawa Hospital, Ottawa, Canada; Clinical Epidemiology Program, Blueprint Translational Research Group, Ottawa Hospital Research Institute, Ottawa, Canada; Regenerative Medicine Program, Ottawa Hospital Research Institute, Ottawa, Canada; 14 Department of Ophthalmology, School of Medicine, University of Colorado Denver, Denver, Colorado, United States; Department of Epidemiology, Johns Hopkins Bloomberg School of Public Health, Baltimore, Maryland, United States of America; 15 Division of Headache, Department of Neurology, Brigham and Women’s Hospital, Harvard Medical School, Boston, Massachusetts, USA; Head of Research, *The BMJ*, London, United Kingdom; 16 Department of Epidemiology and Biostatistics, Indiana University School of Public Health-Bloomington, Bloomington, Indiana, United States of America; 17 Population Health Sciences, Bristol Medical School, University of Bristol, Bristol, United Kingdom; 18 Centre for Reviews and Dissemination, University of York, York, United Kingdom; 19 EPPI-Centre, UCL Social Research Institute, University College London, London, United Kingdom; 20 Li Ka Shing Knowledge Institute of St. Michael’s Hospital, Unity Health Toronto, Toronto, Canada; Epidemiology Division of the Dalla Lana School of Public Health and the Institute of Health Management, Policy, and Evaluation, University of Toronto, Toronto, Canada; Queen’s Collaboration for Health Care Quality Joanna Briggs Institute Centre of Excellence, Queen’s University, Kingston, Canada; 21 Methods Centre, Bruyère Research Institute, Ottawa, Ontario, Canada; School of Epidemiology and Public Health, Faculty of Medicine, University of Ottawa, Ottawa, Canada; 22 Centre for Journalology, Clinical Epidemiology Program, Ottawa Hospital Research Institute, Ottawa, Canada; School of Epidemiology and Public Health, Faculty of Medicine, University of Ottawa, Ottawa, Canada

## Abstract

Matthew Page and co-authors describe PRISMA 2020, an updated reporting guideline for systematic reviews and meta-analyses.

*The Preferred Reporting Items for Systematic reviews and Meta-Analyses (PRISMA) statement, published in 2009, was designed to help systematic reviewers transparently report why the review was done, what the authors did, and what they found. Over the past decade, advances in systematic review methodology and terminology have necessitated an update to the guideline. The PRISMA 2020 statement replaces the 2009 statement and includes new reporting guidance that reflects advances in methods to identify, select, appraise, and synthesise studies. The structure and presentation of the items have been modified to facilitate implementation. In this article, we present the PRISMA 2020 27-item checklist, an expanded checklist that details reporting recommendations for each item, the PRISMA 2020 abstract checklist, and the revised flow diagrams for original and updated reviews*.

Systematic reviews serve many critical roles. They can provide syntheses of the state of knowledge in a field, from which future research priorities can be identified; they can address questions that otherwise could not be answered by individual studies; they can identify problems in primary research that should be rectified in future studies; and they can generate or evaluate theories about how or why phenomena occur. Systematic reviews therefore generate various types of knowledge for different users of reviews (such as patients, healthcare providers, researchers, and policy makers).[1,2]To ensure a systematic review is valuable to users, authors should prepare a transparent, complete, and accurate account of why the review was done, what they did (such as how studies were identified and selected) and what they found (such as characteristics of contributing studies and results of meta-analyses). Up-to-date reporting guidance facilitates authors achieving this.[3]

The Preferred Reporting Items for Systematic reviews and Meta-Analyses (PRISMA) statement published in 2009 (hereafter referred to as PRISMA 2009)[4–10] is a reporting guideline designed to address poor reporting of systematic reviews.[11] The PRISMA 2009 statement comprised a checklist of 27 items recommended for reporting in systematic reviews and an “explanation and elaboration” paper[12–16] providing additional reporting guidance for each item, along with exemplars of reporting. The recommendations have been widely endorsed and adopted, as evidenced by its co-publication in multiple journals, citation in over 60 000 reports (Scopus, August 2020), endorsement from almost 200 journals and systematic review organisations, and adoption in various disciplines. Evidence from observational studies suggests that use of the PRISMA 2009 statement is associated with more complete reporting of systematic reviews,[17–20] although more could be done to improve adherence to the guideline.[21]

Many innovations in the conduct of systematic reviews have occurred since publication of the PRISMA 2009 statement. For example, technological advances have enabled the use of natural language processing and machine learning to identify relevant evidence,[22–24] methods have been proposed to synthesise and present findings when meta-analysis is not possible or appropriate,[25–27] and new methods have been developed to assess the risk of bias in results of included studies.[28,29] Evidence on sources of bias in systematic reviews has accrued, culminating in the development of new tools to appraise the conduct of systematic reviews.[30, 31] Terminology used to describe particular review processes has also evolved, as in the shift from assessing “quality” to assessing “certainty” in the body of evidence.[32] In addition, the publishing landscape has transformed, with multiple avenues now available for registering and disseminating systematic review protocols,[33, 34] disseminating reports of systematic reviews, and sharing data and materials, such as preprint servers and publicly accessible repositories. To capture these advances in the reporting of systematic reviews necessitated an update to the PRISMA 2009 statement.

**Summary points**

- To ensure a systematic review is valuable to users, authors should prepare a transparent, complete, and accurate account of why the review was done, what they did, and what they found
- The PRISMA 2020 statement provides updated reporting guidance for systematic reviews that reflects advances in methods to identify, select, appraise, and synthesise studies
- The PRISMA 2020 statement consists of a 27-item checklist, an expanded checklist that details reporting recommendations for each item, the PRISMA 2020 abstract checklist, and revised flow diagrams for original and updated reviews
- We anticipate that the PRISMA 2020 statement will benefit authors, editors, and peer reviewers of systematic reviews, and different users of reviews, including guideline developers, policy makers, healthcare providers, patients, and other stakeholders

## Development of PRISMA 2020

A complete description of the methods used to develop PRISMA 2020 is available elsewhere.[35] We identified PRISMA 2009 items that were often reported incompletely by examining the results of studies investigating the transparency of reporting of published reviews.[17, 21, 36, 37] We identified possible modifications to the PRISMA 2009 statement by reviewing 60 documents providing reporting guidance for systematic reviews (including reporting guidelines, handbooks, tools, and meta-research studies).[38] These reviews of the literature were used to inform the content of a survey with suggested possible modifications to the 27 items in PRISMA 2009 and possible additional items. Respondents were asked whether they believed we should keep each PRISMA 2009 item as is, modify it, or remove it, and whether we should add each additional item. Systematic review methodologists and journal editors were invited to complete the online survey (110 of 220 invited responded). We discussed proposed content and wording of the PRISMA 2020 statement, as informed by the review and survey results, at a 21-member, two-day, in-person meeting in September 2018 in Edinburgh, Scotland. Throughout 2019 and 2020, we circulated an initial draft and five revisions of the checklist and explanation and elaboration paper to co-authors for feedback. In April 2020, we invited 22 systematic reviewers who had expressed interest in providing feedback on the PRISMA 2020 checklist to share their views (via an online survey) on the layout and terminology used in a preliminary version of the checklist. Feedback was received from 15 individuals and considered by the first author, and any revisions deemed necessary were incorporated before the final version was approved and endorsed by all co-authors.

## The PRISMA 2020 statement

### Scope of the guideline

The PRISMA 2020 statement has been designed primarily for systematic reviews of studies that evaluate the effects of health interventions, irrespective of the design of the included studies. However, the checklist items are applicable to reports of systematic reviews evaluating other interventions (such as social or educational interventions), and many items are applicable to systematic reviews with objectives other than evaluating interventions (such as evaluating aetiology, prevalence, or prognosis). PRISMA 2020 is intended for use in systematic reviews that include synthesis (such as pairwise meta-analysis or other statistical synthesis methods) or do not include synthesis (for example, because only one eligible study is identified). The PRISMA 2020 items are relevant for mixed-methods systematic reviews (which include quantitative and qualitative studies), but reporting guidelines addressing the presentation and synthesis of qualitative data should also be consulted.[39, 40] PRISMA 2020 can be used for original systematic reviews, updated systematic reviews, or continually updated (“living”) systematic reviews. However, for updated and living systematic reviews, there may be some additional considerations that need to be addressed. Where there is relevant content from other reporting guidelines, we reference these guidelines within the items in the explanation and elaboration paper [41] (such as PRISMA-Search [42] in items 6 and 7, Synthesis without meta-analysis (SWiM) reporting guideline [27] in item 13d). Box 1 includes a glossary of terms used throughout the PRISMA 2020 statement.

**Box 1. Glossary of terms**

*Systematic review*—A review that uses explicit, systematic methods to collate and synthesise findings of studies that address a clearly formulated question [43]
*Statistical synthesis*—The combination of quantitative results of two or more studies. This encompasses meta-analysis of effect estimates (described below) and other methods, such as combining P values, calculating the range and distribution of observed effects, and vote counting based on the direction of effect (see McKenzie and Brennan [25] for a description of each method)
*Meta-analysis of effect estimates*—A statistical technique used to synthesise results when study effect estimates and their variances are available, yielding a quantitative summary of results [25]
*Outcome*—An event or measurement collected for participants in a study (such as quality of life, mortality)
*Result*—The combination of a point estimate (such as a mean difference, risk ratio, or proportion) and a measure of its precision (such as a confidence/credible interval) for a particular outcome
*Report*—A document (paper or electronic) supplying information about a particular study. It could be a journal article, preprint, conference abstract, study register entry, clinical study report, dissertation, unpublished manuscript, government report, or any other document providing relevant information
*Record*—The title or abstract (or both) of a report indexed in a database or website (such as a title or abstract for an article indexed in Medline). Records that refer to the same report (such as the same journal article) are “duplicates”; however, records that refer to reports that are merely similar (such as a similar abstract submitted to two different conferences) should be considered unique.
*Study*—An investigation, such as a clinical trial, that includes a defined group of participants and one or more interventions and outcomes. A “study” might have multiple reports. For example, reports could include the protocol, statistical analysis plan, baseline characteristics, results for the primary outcome, results for harms, results for secondary outcomes, and results for additional mediator and moderator analyses

PRISMA 2020 is not intended to guide systematic review conduct, for which comprehensive resources are available.[43–46] However, familiarity with PRISMA 2020 is useful when planning and conducting systematic reviews to ensure that all recommended information is captured. PRISMA 2020 should not be used to assess the conduct or methodological quality of systematic reviews; other tools exist for this purpose.[30, 31] Furthermore, PRISMA 2020 is not intended to inform the reporting of systematic review protocols, for which a separate statement is available (PRISMA for Protocols (PRISMA-P) 2015 statement [47,48]). Finally, extensions to the PRISMA 2009 statement have been developed to guide reporting of network meta-analyses,[49] meta-analyses of individual participant data,[50] systematic reviews of harms, [51] systematic reviews of diagnostic test accuracy studies, [52] and scoping reviews [53]; for these types of reviews we recommend authors report their review in accordance with the recommendations in PRISMA 2020 along with the guidance specific to the extension.

### How to use PRISMA 2020

**Fig 1:**
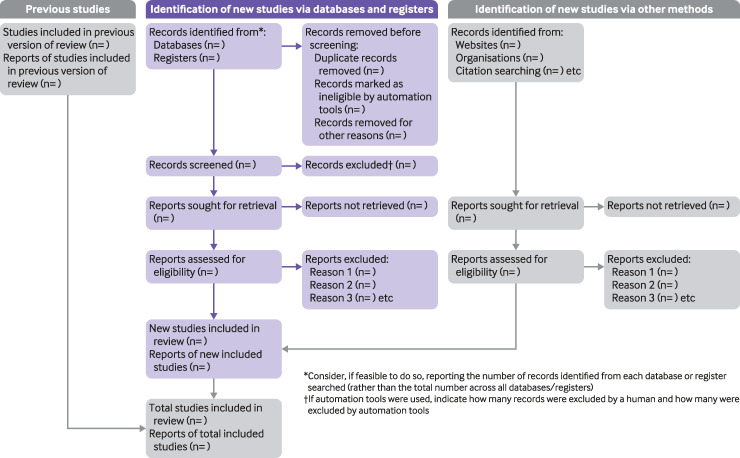
PRISMA 2020 flow diagram template for systematic reviews. The new design is adapted from flow diagrams proposed by Boers,[55] Mayo-Wilson et al.[56] and Stovold et al.[57] The boxes in grey should only be completed if applicable; otherwise they should be removed from the flow diagram. Note that a “report” could be a journal article, preprint, conference abstract, study register entry, clinical study report, dissertation, unpublished manuscript, government report or any other document providing relevant information.

The PRISMA 2020 statement (including the checklists, explanation and elaboration, and flow diagram) replaces the PRISMA 2009 statement, which should no longer be used. Box 2 summarises noteworthy changes from the PRISMA 2009 statement. The PRISMA 2020 checklist includes seven sections with 27 items, some of which include sub-items (Table 1). A checklist for journal and conference abstracts for systematic reviews is included in PRISMA 2020. This abstract checklist is an update of the 2013 PRISMA for Abstracts statement,[54] reflecting new and modified content in PRISMA 2020 (Table 2). A template PRISMA flow diagram is provided, which can be modified depending on whether the systematic review is original or updated (Fig 1).

**Table 1:** PRISMA 2020 item checklist.

Section and topic	Item #	Checklist item	Location where item is reported
**Title**			
Title	1	Identify the report as a systematic review.	
**Abstract**			
Abstract	2	See the PRISMA 2020 for Abstracts checklist (Table 2).	
**Introduction**			
Rationale	3	Describe the rationale for the review in the context of existing knowledge.	
Objectives	4	Provide an explicit statement of the objective(s) or question(s) the review addresses.	
**Methods**			
Eligibility criteria	5	Specify the inclusion and exclusion criteria for the review and how studies were grouped for the syntheses.	
Information sources	6	Specify all databases, registers, websites, organisations, reference lists and other sources searched or consulted to identify studies. Specify the date when each source was last searched or consulted.	
Search strategy	7	Present the full search strategies for all databases, registers and websites, including any filters and limits used.	
Selection process	8	Specify the methods used to decide whether a study met the inclusion criteria of the review, including how many reviewers screened each record and each report retrieved, whether they worked independently, and if applicable, details of automation tools used in the process.	
Data collection process	9	Specify the methods used to collect data from reports, including how many reviewers collected data from each report, whether they worked independently, any processes for obtaining or confirming data from study investigators, and if applicable, details of automation tools used in the process.	
Data items	10a	List and define all outcomes for which data were sought. Specify whether all results that were compatible with each outcome domain in each study were sought (e.g. for all measures, time points, analyses), and if not, the methods used to decide which results to collect.	
	10b	List and define all other variables for which data were sought (e.g. participant and intervention characteristics, funding sources). Describe any assumptions made about any missing or unclear information.	
Study risk of bias assessment	11	Specify the methods used to assess risk of bias in the included studies, including details of the tool(s) used, how many reviewers assessed each study and whether they worked independently, and if applicable, details of automation tools used in the process.	
Effect measures	12	Specify for each outcome the effect measure(s) (e.g. risk ratio, mean difference) used in the synthesis or presentation of results.	
Synthesis methods	13a	Describe the processes used to decide which studies were eligible for each synthesis (e.g. tabulating the study intervention characteristics and comparing against the planned groups for each synthesis (item #5)).	
	13b	Describe any methods required to prepare the data for presentation or synthesis, such as handling of missing summary statistics, or data conversions.	
	13c	Describe any methods used to tabulate or visually display results of individual studies and syntheses.	
	13d	Describe any methods used to synthesise results and provide a rationale for the choice(s). If meta-analysis was performed, describe the model(s), method(s) to identify the presence and extent of statistical heterogeneity, and software package(s) used.	
	13e	Describe any methods used to explore possible causes of heterogeneity among study results (e.g. subgroup analysis, meta-regression).	
	13f	Describe any sensitivity analyses conducted to assess robustness of the synthesised results.	
Reporting bias assessment	14	Describe any methods used to assess risk of bias due to missing results in a synthesis (arising from reporting biases).	
Certainty assessment	15	Describe any methods used to assess certainty (or confidence) in the body of evidence for an outcome.	
**Results**			
Study selection	16a	Describe the results of the search and selection process, from the number of records identified in the search to the number of studies included in the review, ideally using a flow diagram (see Fig 1).	
	16b	Cite studies that might appear to meet the inclusion criteria, but which were excluded, and explain why they were excluded.	
Study characteristics	17	Cite each included study and present its characteristics.	
Risk of bias in studies	18	Present assessments of risk of bias for each included study.	
Results of individual studies	19	For all outcomes, present, for each study: (a) summary statistics for each group (where appropriate) and (b) an effect estimate and its precision (e.g. confidence/credible interval), ideally using structured tables or plots.	
Results of syntheses	20a	For each synthesis, briefly summarise the characteristics and risk of bias among contributing studies.	
	20b	Present results of all statistical syntheses conducted. If meta-analysis was done, present for each the summary estimate and its precision (e.g. confidence/credible interval) and measures of statistical heterogeneity. If comparing groups, describe the direction of the effect.	
	20c	Present results of all investigations of possible causes of heterogeneity among study results.	
	20d	Present results of all sensitivity analyses conducted to assess the robustness of the synthesised results.	
Reporting biases	21	Present assessments of risk of bias due to missing results (arising from reporting biases) for each synthesis assessed.	
Certainty of evidence	22	Present assessments of certainty (or confidence) in the body of evidence for each outcome assessed.	
**Discussion**			
Discussion	23a	Provide a general interpretation of the results in the context of other evidence.	
	23b	Discuss any limitations of the evidence included in the review.	
	23c	Discuss any limitations of the review processes used.	
	23d	Discuss implications of the results for practice, policy, and future research.	
**Other information**			
Registration and protocol	24a	Provide registration information for the review, including register name and registration number, or state that the review was not registered.	
	24b	Indicate where the review protocol can be accessed, or state that a protocol was not prepared.	
	24c	Describe and explain any amendments to information provided at registration or in the protocol.	
Support	25	Describe sources of financial or non-financial support for the review, and the role of the funders or sponsors in the review.	
Competing interests	26	Declare any competing interests of review authors.	
Availability of data, code, and other materials	27	Report which of the following are publicly available and where they can be found: template data collection forms; data extracted from included studies; data used for all analyses; analytic code; any other materials used in the review.	

**Table 2:** PRISMA 2020 for Abstracts checklist*.

Section and topic	Item #	Checklist item
**Title**		
Title	1	Identify the report as a systematic review.
**Background**		
Objectives	2	Provide an explicit statement of the main objective(s) or question(s) the review addresses.
**Methods**		
Eligibility criteria	3	Specify the inclusion and exclusion criteria for the review.
Information sources	4	Specify the information sources (e.g. databases, registers) used to identify studies and the date when each was last searched.
Risk of bias	5	Specify the methods used to assess risk of bias in the included studies.
Synthesis of results	6	Specify the methods used to present and synthesise results.
**Results**		
Included studies	7	Give the total number of included studies and participants and summarise relevant characteristics of studies.
Synthesis of results	8	Present results for main outcomes, preferably indicating the number of included studies and participants for each. If meta-analysis was done, report the summary estimate and confidence/credible interval. If comparing groups, indicate the direction of the effect (i.e. which group is favoured).
**Discussion**		
Limitations of evidence	9	Provide a brief summary of the limitations of the evidence included in the review (e.g. study risk of bias, inconsistency and imprecision).
Interpretation	10	Provide a general interpretation of the results and important implications.
**Other**		
Funding	11	Specify the primary source of funding for the review.
Registration	12	Provide the register name and registration number.

*This abstract checklist retains the same items as those included in the PRISMA for Abstracts statement published in 2013, [54] but has been revised to make the wording consistent with the PRISMA 2020 statement and includes a new item recommending authors specify the methods used to present and synthesise results (item #6).

**Box 2. Noteworthy changes to the PRISMA 2009 statement**

- Inclusion of the abstract reporting checklist within PRISMA 2020 (see item #2 and Table 2).
- Movement of the ‘Protocol and registration’ item from the start of the Methods section of the checklist to a new Other section, with addition of a sub-item recommending authors describe amendments to information provided at registration or in the protocol (see item #24a-24c).
- Modification of the ‘Search’ item to recommend authors present full search strategies for *all* databases, registers and websites searched, not just at least one database (see item #7).
- Modification of the ‘Study selection’ item in the Methods section to emphasise the reporting of how many reviewers screened each record and each report retrieved, whether they worked independently, and if applicable, details of automation tools used in the process (see item #8).
- Addition of a sub-item to the ‘Data items’ item recommending authors report how outcomes were defined, which results were sought, and methods for selecting a subset of results from included studies (see item #10a).
- Splitting of the ‘Synthesis of results’ item in the Methods section into six sub-items recommending authors describe: the processes used to decide which studies were eligible for each synthesis; any methods required to prepare the data for synthesis; any methods used to tabulate or visually display results of individual studies and syntheses; any methods used to synthesise results; any methods used to explore possible causes of heterogeneity among study results (such as subgroup analysis, meta-regression); and any sensitivity analyses used to assess robustness of the synthesised results (see item #13a-13f).
- Addition of a sub-item to the ‘Study selection’ item in the Results section recommending authors cite studies that might appear to meet the inclusion criteria, but which were excluded, and explain why they were excluded (see item #16b).
- Splitting of the ‘Synthesis of results’ item in the Results section into four sub-items recommending authors: briefly summarise the characteristics and risk of bias among studies contributing to the synthesis; present results of all statistical syntheses conducted; present results of any investigations of possible causes of heterogeneity among study results; and present results of any sensitivity analyses (see item #20a-20d).
- Addition of new items recommending authors report methods for and results of an assessment of certainty (or confidence) in the body of evidence for an outcome (see items #15 and #22).
- Addition of a new item recommending authors declare any competing interests (see item #26).
- Addition of a new item recommending authors indicate whether data, analytic code and other materials used in the review are publicly available and if so, where they can be found (see item #27).

We recommend authors refer to PRISMA 2020 early in the writing process, because prospective consideration of the items may help to ensure that all the items are addressed. To help keep track of which items have been reported, the PRISMA statement website (http://www.prisma-statement.org/) includes fillable templates of the checklists to download and complete (also available in S1 PRISMA 2020 checklist). We have also created a web application that allows users to complete the checklist via a user-friendly interface [58] (available at https://prisma.shinyapps.io/checklist/ and adapted from the Transparency Checklist app [59]). The completed checklist can be exported to Word or PDF. Editable templates of the flow diagram can also be downloaded from the PRISMA statement website.

We have prepared an updated explanation and elaboration paper, in which we explain why reporting of each item is recommended and present bullet points that detail the reporting recommendations (which we refer to as elements).[41] The bullet-point structure is new to PRISMA 2020 and has been adopted to facilitate implementation of the guidance.[60, 61] An expanded checklist, which comprises an abridged version of the elements presented in the explanation and elaboration paper, with references and some examples removed, is available in S2 PRISMA 2020 expanded checklist. Consulting the explanation and elaboration paper is recommended if further clarity or information is required.

Journals and publishers might impose word and section limits, and limits on the number of tables and figures allowed in the main report. In such cases, if the relevant information for some items already appears in a publicly accessible review protocol, referring to the protocol may suffice. Alternatively, placing detailed descriptions of the methods used or additional results (such as for less critical outcomes) in supplementary files is recommended. Ideally, supplementary files should be deposited to a general-purpose or institutional open-access repository that provides free and permanent access to the material (such as Open Science Framework, Dryad, figshare). A reference or link to the additional information should be included in the main report. Finally, although PRISMA 2020 provides a template for where information might be located, the suggested location should not be seen as prescriptive; the guiding principle is to ensure the information is reported.

## Discussion

Use of PRISMA 2020 has the potential to benefit many stakeholders. Complete reporting allows readers to assess the appropriateness of the methods, and therefore the trustworthiness of the findings. Presenting and summarising characteristics of studies contributing to a synthesis allows healthcare providers and policy makers to evaluate the applicability of the findings to their setting. Describing the certainty in the body of evidence for an outcome and the implications of findings should help policy makers, managers, and other decision makers formulate appropriate recommendations for practice or policy. Complete reporting of all PRISMA 2020 items also facilitates replication and review updates, as well as inclusion of systematic reviews in overviews (of systematic reviews) and guidelines, so teams can leverage work that is already done and decrease research waste.[36, 62, 63]

We updated the PRISMA 2009 statement by adapting the EQUATOR Network’s guidance for developing health research reporting guidelines.[64] We evaluated the reporting completeness of published systematic reviews,[17, 21, 36, 37] reviewed the items included in other documents providing guidance for systematic reviews,[38] surveyed systematic review methodologists and journal editors for their views on how to revise the original PRISMA statement,[35] discussed the findings at an in-person meeting, and prepared this document through an iterative process. Our recommendations are informed by the reviews and survey conducted before the in-person meeting, theoretical considerations about which items facilitate replication and help users assess the risk of bias and applicability of systematic reviews, and co-authors’ experience with authoring and using systematic reviews.

Various strategies to increase the use of reporting guidelines and improve reporting have been proposed. They include educators introducing reporting guidelines into graduate curricula to promote good reporting habits of early career scientists [65]; journal editors and regulators endorsing use of reporting guidelines [18]; peer reviewers evaluating adherence to reporting guidelines [61, 66]; journals requiring authors to indicate where in their manuscript they have adhered to each reporting item[67]; and authors using online writing tools that prompt complete reporting at the writing stage.[60] Multi-pronged interventions, where more than one of these strategies are combined, may be more effective (such as completion of checklists coupled with editorial checks).[68] However, of 31 interventions proposed to increase adherence to reporting guidelines, the effects of only 11 have been evaluated, mostly in observational studies at high risk of bias due to confounding.[69] It is therefore unclear which strategies should be used. Future research might explore barriers and facilitators to the use of PRISMA 2020 by authors, editors, and peer reviewers, designing interventions that address the identified barriers, and evaluating those interventions using randomised trials. To inform possible revisions to the guideline, it would also be valuable to conduct think-aloud studies [70] to understand how systematic reviewers interpret the items, and reliability studies to identify items where there is varied interpretation of the items.

We encourage readers to submit evidence that informs any of the recommendations in PRISMA 2020 (via the PRISMA statement website: http://www.prisma-statement.org/). To enhance accessibility of PRISMA 2020, several translations of the guideline are under way (see available translations at the PRISMA statement website). We encourage journal editors and publishers to raise awareness of PRISMA 2020 (for example, by referring to it in journal “Instructions to authors”), endorsing its use, advising editors and peer reviewers to evaluate submitted systematic reviews against the PRISMA 2020 checklists, and making changes to journal policies to accommodate the new reporting recommendations. We recommend existing PRISMA extensions [47, 49–53, 71, 72] be updated to reflect PRISMA 2020 and advise developers of new PRISMA extensions to use PRISMA 2020 as the foundation document.

### Conclusion

We anticipate that the PRISMA 2020 statement will benefit authors, editors, and peer reviewers of systematic reviews, and different users of reviews, including guideline developers, policy makers, healthcare providers, patients, and other stakeholders. Ultimately, we hope that uptake of the guideline will lead to more transparent, complete, and accurate reporting of systematic reviews, thus facilitating evidence based decision making.

## Supporting information

S1 PRISMA 2020 checklist (PDF)

S2 PRISMA 2020 expanded checklist (PDF)
